# Web-Based Health Information–Seeking Methods and Time Since Provider Engagement: Cross-sectional Study

**DOI:** 10.2196/42126

**Published:** 2022-11-30

**Authors:** Eden Shaveet, Catherine Urquhart, Marissa Gallegos, Olaf Dammann, Laura Corlin

**Affiliations:** 1 Department of Public Health and Community Medicine, Tufts University School of Medicine Boston, MA United States; 2 Department of Community Health Tufts University School of Arts and Sciences Medford, MA United States; 3 Department of Gynecology and Obstetrics Hannover Medical School Hannover Germany; 4 Department of Civil and Environmental Engineering Tufts University School of Engineering Medford, MA United States

**Keywords:** internet, social media, information-seeking behavior, consumer health information, physician-patient relations, trust

## Abstract

**Background:**

The use of web-based methods to seek health information is increasing in popularity. As web-based health information (WHI)–seeking affects health-related decision support and chronic symptom self-management, WHI-seeking from online sources may impact health care decisions and outcomes, including care-seeking decisions. Patients who are routinely connected to physicians are more likely to receive better and more consistent care. Little is known about whether WHI-seeking impacts the frequency at which patients engage with health care providers.

**Objective:**

Our primary objective was to describe the associations between the use of web-based methods to seek information about one’s own health and the time since last engaging with a health care provider about one’s own health. Additionally, we aimed to assess participants’ trust in health care organizations to contextualize our findings.

**Methods:**

We analyzed data from US adults participating in the nationally representative Tufts Equity in Health, Wealth, and Civic Engagement Survey (N=1034). Bivariate associations between demographic characteristics and health information–seeking methods were assessed with Pearson chi-squared tests. Bivariate associations of Medical Mistrust Index (MMI) scores with each health information–seeking method and time since provider engagement were assessed with *F* tests and adjusted Wald tests. We fit a multivariable logistic regression model to assess the association between WHI-seeking within the 12 months prior to survey (alone or in combination with provider-based methods versus provider only) and engagement with a provider more than 1 year prior to the time of survey, adjusting for age, race and ethnicity, sex, education, insurance coverage, and MMI.

**Results:**

Age, race and ethnicity, educational attainment, health insurance source, MMI, and time since provider engagement were each significantly associated with the health information–seeking method in bivariate analyses. Compared to using only provider-based health information seeking methods, WHI-based methods alone or in combination with provider-based methods were associated with a 51% lower likelihood (odds ratio 0.49, 95% CI 0.27-0.87) of engaging with a provider within the previous year. Participants who used WHI-seeking methods alone and those who had not engaged with a health care provider within the previous year demonstrated a higher mean MMI score; however, MMI was not a significant predictor of time since engagement with a provider in the multivariable analysis.

**Conclusions:**

Our findings from a nationally representative survey suggest that for those who use WHI-seeking methods (alone or in combination with provider-based information-seeking methods), there is a statistically significant lower likelihood of engaging with a provider in a year compared to those who only use provider-based methods. Future research should consider the intent of a person’s visit with a provider, trust in health care systems, methods of provider engagement, and specific web-based platforms for health information.

## Introduction

Routine engagement with health care providers is vital for the early detection and treatment of disease, as well as for preventive care delivery [1,2]. Most often achieved through interaction with primary care services, routine provider engagement is essential for the maintenance of individual and community health [3]. Patients who are routinely connected to physicians are more likely to receive better and more consistent care [3,4].

Barriers to accessing routine or specialty health care, such as lack of health insurance coverage, are associated with poor health outcomes [5,6]. It has been suggested that such barriers may prompt online health information–seeking behaviors for self-management [7].

Health information–seeking methods are the means by which individuals acquire information about their health, health promotion, health risks, and illness [8]. Use of internet-enabled technologies to seek health information, often referred to as web-based health information (WHI)–seeking methods, has increased exponentially across US populations since the early 2000s [9] and notably during the COVID-19 pandemic, which began at the close of 2019 [10]. Use of web-based sources to obtain health information may have considerable effects on individuals’ health care decisions and outcomes [9]. These effects may include encouraging use of ambulatory services, informing decisions to self-manage symptoms, and influencing attitudes toward a disease, treatment, or procedure [9,11,12].

Prior work investigating the impact of WHI-seeking on patient-physician relationships suggested that WHI-seeking may improve relationships by promoting meaningful patient participation in conversation during appointments [13]. Other work examining the impact of WHI-seeking on patient treatment compliance concluded that encouraging patients to seek health information relating to their treatment online may improve overall compliance with recommendations [14]. However, contrary to work positing that WHI-seeking enables meaningful patient engagement with health care providers [13], other work has suggested that participation in online forums may promote mistrust in health services [15].

In considering the role of WHI-seeking on patient-provider relationships and patient well-being, it can be informative to understand how this affects the frequency at which patients engage with health care providers. Prior research suggests that WHI-seeking may lead to more frequent visits with physicians and that trust in information from health care providers may moderate this association [16,17]. Nevertheless, as WHI-seeking behaviors have increased, little research has examined its role in patient engagement with health care providers [18]. This paper is an attempt to fill this gap in the literature. Specifically, using nationally representative data, we seek to explore and generate hypotheses regarding the association between WHI-seeking behavior and length of time since last engaging with a health care provider about ones’ own health.

## Methods

### Study Sample

Participants were recruited via a random sampling of telephone numbers and residential addresses by Ipsos, a multinational market research and social science company. As described previously, data were collected via the second deployment of the Equity in Health, Wealth, and Civic Engagement Survey designed by Tufts University [19]. The survey was fielded in English and Spanish, and was deployed between April 23, 2021, and May 3, 2021, through the Ipsos KnowledgePanel, an online, nationally representative, probability-based panel used to retrieve insights from US adults. Upon accepting the initial invitation to join the KnowledgePanel, respondents were asked to complete a short demographic survey prior to becoming active KnowledgePanel members. Eligible participants for the Equity in Health, Wealth, and Civic Engagement Survey were noninstitutionalized US adults aged 18 years and older who were proficient in English or Spanish languages. Those without access to the internet were provided a laptop and internet access by Ipsos at no cost.

### Ethics Approval

All study protocols were reviewed and approved by the Social, Behavioral, and Educational Research Institutional Review Board at Tufts University, Boston, MA (protocol STUDY00000428). All participants provided informed consent to participate in the Ipsos KnowledgePanel and to complete the Tufts Equity in Health, Wealth, and Civic Engagement Survey. Participants received standard incentive payments upon survey completion (eg, 1000 points, the cash-equivalent of US $1 and an entry into the KnowledgePanel sweepstakes for completing a survey that takes longer than 15 minutes; median completion time=15 minutes). Participant responses were deidentified, and precautions were taken to ensure confidentiality and privacy for participants (eg, storing data only on secure drives) [19].

### Procedures

Of the 2107 KnowledgePanel participants invited to complete the survey, 1449 (68.77%) responded to the Tufts Equity in Health, Wealth, and Civic Engagement Survey. Participants included in our analysis (n=1034) were those who met both of the following inclusion criteria: (1) provided information about the time elapsed since they had last engaged with a health care provider about their own health, and (2) indicated they had used web-based or health care provider–based methods to seek health information in the prior year, as described in our Measures subsection.

### Measures

#### Demographic Variables

Demographic and contextual items incorporated in our analyses included self-reported age (18-24, 25-34, 35-44, 45-54, 55-64, 65-75, or >75 years), race and ethnicity (Hispanic, non-Hispanic Black, non-Hispanic White, or non-Hispanic multiracial/other), sex (female or male), educational attainment (less than high school diploma, high school diploma/General Educational Development, some college/associate’s degree, bachelor’s degree, or master’s degree/higher), annual household income (<US $10,000, US $10,000-24,999, US $25,000-49,000, US $50,000-74,999, US $75,000-99,999, US $100,000-149,999, or ≥ US $150,000), insurance coverage (no insurance, employer-sponsored, government-sponsored [including Medicare, Medicaid, and military], health insurance marketplace, or other), and household internet access (defined as 1 or more members of a participant’s household, including themselves, having access to the internet).

#### Health Information–Seeking Variables

Our health information–seeking variables were derived from a survey item (“Have you used any of the following sources for health information in the past 12 months?”) in which participants could select all responses that applied (“Doctor,” “Pharmacist,” “Nurse, nurse practitioner or physician’s assistant,” “Relative, friend, or co-worker,” “Someone you know who has a particular medical condition,” “Disease-related association or society,” “Patient support group or foundation,” “Educational forum at a local clinic, hospital, community center or other location,” “Pharmaceutical company,” “Health insurance company,” “Newspapers or magazines,” “Television,” “The internet,” “Social Media [such as Facebook, Twitter]”, “Healthcare app for smartphone or tablet”). Affirmative responses of “The internet,” “Social media,” or “Healthcare app” were combined into a composite variable indicating the use of WHI-seeking methods. Affirmative responses of “Doctor,” or “Nurse, nurse practitioner or physician's assistant” were combined into a composite variable indicating provider-based health information–seeking.

#### Time-Since-Provider-Engagement Variables

We developed a dichotomous variable for time since health care provider engagement based on participant responses to *“*How long has it been since you last saw or talked to a doctor or other healthcare professional about your own health?” Response options included “6 months or less,” “More than 6 months, but not more than 1 year ago,” “More than 1 year, but not more than 2 years ago,” “More than 2 years, but not more than 5 years ago,” “More than 5 years ago,” and “Never.” We chose to make this variable dichotomous (≤1 year or >1 year ago) for sample size reasons, but the full distribution of responses is shown in Multimedia Appendix 1, Table S1.

#### Medical Mistrust Index Variables

To aid in the interpretation of our descriptive results, we examined scores on the Medical Mistrust Index (MMI) developed to assess a participant’s trust in health care organizations [20]. The MMI is a 7-item index offered on a 5-point Likert scale including the following response values: “strongly disagree,” “moderately disagree,” “neutral,” “moderately agree,” and “strongly agree” [20]. We coded responses from 0 (“strongly disagree”) to 4 (“strongly agree”). Total scores could be between 0 and 28, with higher values reflecting greater perceived mistrust of health care organizations. The seven index items include the following: (1) “You’d better be cautious when dealing with healthcare organizations.”(2)“Patients have sometimes been deceived or misled by healthcare organizations.” (3) “When healthcare organizations make mistakes, they usually cover it up.” (4) “Healthcare organizations have sometimes done harmful experiments on patients without their knowledge.” (5) “Healthcare organizations don’t always keep your information totally private.”(6) “Sometimes I wonder if healthcare organizations really know what they are doing.” (7) “Mistakes are common in healthcare organizations.”

### Analysis

We examined demographic characteristics of participants and MMI scores overall by category of health information–seeking method (WHI only, provider-based only, both) and by time since last provider engagement. Bivariate associations between demographic characteristics and health information–seeking method were assessed with Pearson chi-squared tests. Bivariate associations of MMI with each health information–seeking method and time since provider engagement were assessed with *F* tests (for overall associations) and adjusted Wald tests (for pairwise comparisons). These tests were selected based on their ability to support survey weighting. We fit a multivariable logistic regression model to assess the association between WHI-seeking within the 12 months prior to survey (alone or in combination with provider-based methods versus provider only) and engagement with a provider more than 1 year prior to the time of survey, adjusting for age, race and ethnicity, sex, education, insurance coverage (dichotomous; insured or uninsured), and MMI. We chose a multivariable logistic regression model to accommodate our dichotomous outcome and adjust for relevant covariates. These covariates were chosen based on significant bivariate associations with the primary exposure or outcome. All analyses were conducted using Stata 17 (StataCorp) and R 4.1.2 (The R Foundation for Statistical Computing) and applied sample weights to be more representative of the US population based on the US Census Bureau's 2019 current population estimates [19]. Sample weights varied from 0.131 to 4.827 with a median of 0.826 for the full sample.

## Results

### Sample Characteristics

Sample characteristics are described in Table 1. Briefly, most (70.85%) participants were between 25 and 64 years old. Most were non-Hispanic White (63.19%) and approximately half (53.47%) were female. Most participants (68.41%) had more than a high school level of education, and most (56.06%) had an annual household income of at least US $75,000. Nearly all participants (95.42%) had health insurance, and nearly all (99.07%) had internet access. Nearly half of participants (47.20%) used both WHI and provider-based seeking methods, with only 16.96% using only WHI-based methods. Whereas 87.09% of participants had engaged with a health care provider in the previous year, only 83.04% of participants reported provider-based or web- and provider-based information-seeking methods.

**Table 1:** Sample characteristics overall, by health information–seeking method, and by time since provider engagement.

Characteristic			Overall unweighted n (weighted %)		Health information–seeking method (weighted row %)									Time since provider engagement (weighted row %)						
					Web-based only		Provider-based only		Web- and provider-based		*P* value^a^			≤1 year		>1 year		*P* value^a^		
Overall			1034 (100)		16.96		35.84		47.20					87.09		12.91				
**Age in years (N=1034)**												.005							<.001	
	18-24	33 (6.15)		17.33		14.46		68.21					80.29		19.71					
	25-34	122 (18.01)		17.25		35.07		47.68					78.16		21.84					
	35-44	157 (16.87)		19.55		31.65		48.80					81.21		18.79					
	45-54	145 (14.19)		19.46		34.31		46.23					85.99		14.01					
	55-64	255 (21.78)		21.02		34.95		44.03					92.45		7.55					
	65-74	207 (14.12)		10.15		40.68		49.17					95.39		4.61					
	≥75	115 (8.88)		8.02		57.13		34.85					96.49		3.51					
**Race and ethnicity (N=1034)**												.001							.001	
	White, non-Hispanic	505 (63.19)		14.31		36.57		49.12					90.65		9.35					
	Black, non-Hispanic	235 (11.18)		18.09		42.00		39.91					89.61		10.39					
	Hispanic	231 (15.22)		28.07		37.11		34.81					78.14		21.86					
	Other or 2+ races, non-Hispanic	63 (10.42)		15.57		22.94		61.48					75.88		24.12					
**Sex (N=1034)**												.16							.003	
	Female	518 (53.47)		15.35		34.19		50.46					90.99		9.01					
	Male	516 (46.53)		18.80		37.74		43.46					82.61		17.39					
**Education (N=1034)**												.001							.16	
	Less than a high school diploma	90 (8.76)		29.68		33.34		36.98					81.67		18.33					
	High school diploma/GED^b^	258 (22.83)		21.28		41.61		37.11					83.84		16.16					
	Some college or associate’s degree	282 (31.40)		15.04		38.16		46.80					90.93		9.07					
	Bachelor’s degree	230 (21.44)		13.01		34.84		52.14					84.65		15.35					
	Master’s degree or higher	174 (15.58)		12.75		25.51		61.74					90.53		9.47					
**Annual household income (N=1034)**												.34							.63	
	<US $10,000	26 (2.17)		13.05		31.72		55.22					91.56		8.44					
	US $10,000-24,999	93 (8.83)		19.87		38.80		41.33					86.32		13.68					
	US $25,000-$49,999	177 (14.89)		23.58		30.88		45.54					88.67		11.33					
	US $50,000-74,999	173 (18.05)		17.00		33.30		49.69					84.22		15.78					
	US $75,000-99,999	159 (14.69)		20.07		36.32		43.61					82.95		17.05					
	US $100,000-149,999	203 (19.59)		14.01		42.68		43.31					90.07		9.93					
	≥US $150,000	203 (21.78)		12.15		34.08		53.77					88.38		11.62					
**Health insurance source (N=1031)**												<.001							<.001	
	No insurance	54 (4.58)		52.74		20.74		26.51					46.50		53.50					
	Employer	546 (57.39)		15.09		34.65		50.26					87.71		12.29					
	Government^c^	346 (30.02)		10.67		41.91		47.42					93.71		6.29					
	Health insurance marketplace	48 (4.26)		40.41		24.67		34.92					76.17		23.83					
	Other source	37 (3.74)		28.02		29.05		42.92					84.75		15.25					
**Internet access^d^ (N=1029)**												.37							.74	
	Yes	1017 (99.07)		16.97		35.48		47.56					87.14		12.86					
	No	12 (0.93)		15.60		57.81		26.60					83.78		16.22					
Medical Mistrust Index (N = 1034)^e^			14.6 (14.1-15.1)		16.4 (15.3-17.5)		14.3 (13.5-15.2)		14.2 (13.5-14.9)		.002			14.4 (13.9-14.9)		16.1 (14.6-17.6)		.04		
**Time since provider engagement (N=1034)**												<.001								
	≤1 year	910 (87.09)		12.41		38.12		49.47												
	>1 year	124 (12.91)		47.61		20.51		31.88												
**Health information–seeking method (N=1034)**																				<.001
	Web-based only	190 (16.96)		N/A^f^		N/A		N/A					63.75		36.25					
	Provider-based only	388 (35.84)		N/A		N/A		N/A					92.61		7.39					
	Web- and provider-based	456 (47.20)		N/A		N/A		N/A					91.28		8.72					

^a^*P* value for Pearson chi-squared test or *F* test (for Medical Mistrust Index).

^b^GED: General Education Development.

^c^Includes Medicare, Medicaid, and military insurance.

^d^Defined as 1 or more members of a participant’s household having access to the internet.

^e^Values in this row are reported as mean (95% CIs). *P* values are for *F* test.

^f^N/A: not applicable. Corresponding rows and columns refer to the same groups.

### Bivariate Associations

Age, race and ethnicity, educational attainment, health insurance source, MMI, and time since provider engagement were each significantly associated with the health information–seeking methods in bivariate analyses (Table 1). For example, MMI scores were significantly higher for people who used WHI-based methods alone compared to either provider-based methods alone (*P*=.003) or both WHI- and provider-based methods (*P*=.001). Similarly, age, race and ethnicity, sex, MMI, and health insurance source were each significantly associated with time since provider engagement in bivariate analyses (Table 1). For example, MMI scores were significantly higher for people who had not engaged with a provider in the previous year (*P*=.04). However, among those who had used WHI-based methods (alone or in combination with provider-based methods; n=646), there was no significant difference in mean MMI scores (*P*=.08).

### Multivariable Associations

Compared to using only provider-based health information–seeking methods, using WHI-based methods alone or in combination with provider-based methods was associated with a 51% lower likelihood (odds ratio 0.49, 95% CI 0.27-0.87) of engaging with a provider within the previous year (Table 2). Being female or insured was associated with an increased likelihood of engaging with a provider within the previous year, whereas identifying as non-Hispanic and more than 2 races or a race other than White or Black was associated with a lower likelihood (Table 2).

**Table 2:** Association between web-based health information–seeking within the prior year and engagement with a provider more than 1 year prior (N=1031).

			Engagement with a provider within the past year (referent: >1 year) odds ratio (95% CI)
**Health information–seeking (referent: only provider-based information-seeking)**			
	Web-based only or web-based and provider-based	0.49 (0.27, 0.87)^a^	
**Age group (referent: 18-24 years)**			
	25-34 years	0.66 (0.18-2.45)	
	35-44 years	0.85 (0.25-2.92)	
	45-54 years	1.14 (0.31-4.24)	
	55-64 years	2.11 (0.62-7.18)	
	65-74 years	2.87 (0.79-10.41)	
	≥75 years	3.60 (0.82-15.89)	
**Race and ethnicity (referent: non-Hispanic White)**			
	Black, non-Hispanic	1.33 (0.65-2.73)	
	Hispanic	0.63 (0.35-1.12)	
	Other or 2+ races, non-Hispanic	0.36 (0.16-0.84)^a^	
**Sex (referent: male)**			
	Female	2.28 (1.32-3.92)^a^	
**Education (referent: less than high school diploma)**			
	High school diploma/GED^b^	0.63 (0.28-1.45)	
	Some college or associate’s degree	1.21 (0.51-2.90)	
	Bachelor’s degree	0.84 (0.34-2.08)	
	Master’s degree or higher	1.50 (0.54-4.19)	
**Insurance coverage (referent: uninsured)**			
	Insured	5.73 (2.71-12.15)^a^	
Medical Mistrust Index			0.97 (0.93-1.01)

^a^*P*<.05.

^b^GED: General Education Development.

## Discussion

### Principal Results

Our nationally representative cross-sectional study sought to further the discussion about how the use of WHI-seeking methods relate to the time duration since last engaging with a health care provider about one’s own health. We observed that for those who use WHI-seeking methods (alone or in combination with provider-based information-seeking methods), there is a lower likelihood of engaging with a provider in a year compared to those who only use provider-based methods. Given that more than half of US adults use the internet as their primary source of health information [18], our findings, paired with literature suggesting a decline in the frequency at which commercially insured US adults receive primary care [21], point to potentially novel shifts in patient engagement for individuals who seek WHI. These interpretations are consistent with prior work suggesting that WHI-seeking may influence a person’s medical treatment decisions, including whether to visit a physician or not [22]. These interpretations are also consistent with prior work suggesting that WHI-seeking influences patients’ trust in health care providers [23-25], which is in turn associated with the frequency at which patients engage with their providers [26,27]. These findings differ, however, from previous work suggesting that WHI-seeking leads to more frequent visits with physicians [16] and that this effect is larger for those who exhibit lower trust in information offered by health care providers [17].

Medical mistrust remains a complex challenge that inhibits access to care by dissuading utilization of and participation in health services [20]. Recognized as a social determinant of health driving health disparities for marginalized groups [28], medical mistrust reflects the influence of both health misinformation and longstanding struggles to restore trust following historical medical misdeeds and mistakes [29]. Prior work suggests that some WHI-seeking methods may promote patient mistrust in health services by virtue of repeat false or misleading portrayals of health care, such as through online forums or social media posts [15,30].

In our study, statistically significant differences were observed in mean MMI scores by health information method used in the previous year and with time since engagement with a health care provider, with those who used WHI-seeking methods alone and those who had not engaged with a health care provider within the previous year demonstrating a higher mean MMI score. Whereas this may suggest a relationship between use of WHI-seeking methods alone and higher perceived mistrust of health care organizations, MMI was not a significant predictor of time since engagement with a provider in the multivariable analysis. Additional work is needed to identify the role of MMI in the relationship between WHI-seeking and health care provider engagement. Nuances in these relationships—such as differences by specific web-based platforms—may be especially salient given literature suggesting that the availability and use of WHI by patients can be beneficial to patient-provider relationships [13,14,23]. For example, several studies assert that WHI-seeking may promote health literacy among patients and improve communication with providers [13,23]. Others find that physician-encouraged WHI-seeking by patients may improve patient compliance with treatment recommendations [14]. Some works speculate that the impact of WHI-seeking on patient-provider relationships is contingent on several factors, including the quality of WHI retrieved by patients [23,31], the willingness of providers to discuss WHI brought forth by a patient [32], a provider’s reactions to a patient’s presentation of WHI with respect to their treatment [13,33], and whether the WHI serves to complement or challenge a provider’s medical expertise [34].

Lack of health insurance coverage, a well-documented barrier to care, was also a significant predictor of time since engagement with a health care provider. Whereas the plurality of respondents in our sample used a combination of web-based and provider-based methods to seek health information in the previous year, over half of respondents without insurance used web-based methods only and over half had not engaged with a health care provider in the previous year. These findings suggest that barriers to routine care, such as lack of insurance or other financial constraints, might prompt the use of WHI-seeking methods for condition self-management [7,35].

### Limitations and Strengths

Our study had several limitations. First, our cross-sectional survey was fielded during the COVID-19 pandemic, and the results may not be generalizable beyond this time frame. Disruption of routine health care service delivery and escalation of novel service delivery methods throughout the pandemic, such as the increased use of telehealth [36], impacted the frequency and means by which patients engaged with health care providers overall [37]. As respondents were not asked about how they engaged with health care providers, this study does not explicitly specify whether the 87.09% of respondents who engaged with providers used telehealth services or engaged with providers face to face (which could explain the counterintuitive statistic in our sample that 83.04% engaged with provider-based or web- and provider-based information-seeking methods). It is also possible that respondents interpreted response options of “the internet” or “Healthcare app” to include use of telehealth services or patient portals. Second, there might have been social desirability bias in terms due to the self-reported nature of when participants last engaged with health care providers. Third, our sample was limited to individuals who sought WHI or information from provider-based sources in the 12 months prior to the survey and excluded those who only sought health information from other sources during this time frame. Fourth, although participants without access to the internet were provided a laptop and internet connection by Ipsos at no cost, this alone does not guarantee that the sample is adequately representative of those without internet access. Similarly, respondents were recruited via random sampling of telephone numbers and residential addresses. Thus, those without telephone numbers or residential addresses may be underrepresented in our sample. These issues and others might have affected the ability of our survey to be truly nationally representative. Finally, our sample size was relatively small, and we therefore dichotomized certain variables, such as time since provider engagement. We might have lost nuances in trends because of this decision, and our multivariable regression model might have been underpowered.

Despite these limitations, our study presents several strengths, including its sampling methodology coupled with use of sample weights to maximize its representativeness of the US population. Collection of information about health information–seeking methods and engagement with health care providers at this scale offers novel insight and corroborates previously suggested relationships between WHI-seeking behaviors and health care service utilization. It is important to add this current evidence, as patients’ relationships with the internet as a source of health information evolves as societal norms and ways of accessing information change. Our work also provides novel insight into several characteristics that had not been investigated together in relation to WHI-seeking and time since provider engagement, such as insurance coverage. In addition, we were able to assess the role of medical mistrust, a potentially critical factor in the relationship between health information–seeking methods and engagement with health care providers. These findings present key insights for an emerging area of research and offer directions for inquiry in future research.

### Implications and Future Research

The association between use of WHI-seeking methods and time since provider engagement suggests questions for future research. For example, an investigation could be conducted to identify associations between use of WHI-seeking methods and time since provider engagement by service rendered (eg, routine or preventive versus emergency). Whereas delays in provider engagement for routine or preventive care are potentially detrimental to individual and community health, reducing unnecessary emergency provider visits and associated per capita costs is desirable for patients and health systems [38]. Additionally, although the use of WHI-seeking methods has demonstrable benefits [13,14,39,40], concerns arise around exposure to online health misinformation [41]. Misinformation and disinformation around health have received increased public attention in recent years due to the negative impact they have had on individual and public health, notably during the COVID-19 pandemic [42]. Although the popularity of online information and social media platforms has contributed to the prevalence of online misinformation [43], it is unknown whether those who use the internet as a primary source of health information are disproportionately exposed to health misinformation. Future research should investigate whether exposure to health misinformation or other factors underlie the associations we observed between the method of health information–seeking and time since provider engagement.

### Conclusions

Our findings from a nationally representative survey suggest that for those who use WHI-seeking methods (alone or in combination with provider-based information-seeking methods), there is a statistically significant lower likelihood of engaging with a provider in a year compared to those who only use provider-based methods. Future research should consider the intent of a person’s visit with a provider, trust in health care systems, methods of provider engagement, and specific web-based platforms for health information.

## Appendix

Multimedia Appendix 1 Table displaying time since provider engagement for study sample (N=1034; weighted proportions).

## Abbreviations

MMI: Medical Mistrust Index
WHI: web-based health information

## Data Availability

A deidentified version of the study data will be made available upon reasonable request to Peter Levine. Peter Levine is associate dean of academic affairs and Lincoln Filene professor of citizenship and public affairs in Tufts University’s Jonathan Tisch College of Civic Life. He is also a coprincipal investigator of the Equity in Health, Wealth and Civic Engagement Priority Research Cluster. Exploration of some of the salient measures collected in our survey, as well as bivariate comparisons and visualizations of results, are available at our public-facing website.
