# Supplementary material for: Web-Based Health Information–Seeking Methods and Time Since Provider Engagement: Cross-sectional Study
Source: JMIR Form Res. 2022 Nov 30;6(11):e42126. doi: 10.2196/42126 (PMC9752449; doi:10.2196/42126)
Supplement: Multimedia Appendix 1 [file formative_v6i11e42126_app1.docx]

## Appendix 1

***Supplemental Materials***

Supplemental Table 1: Time Since Provider Engagement

| **Time Since Provider Engagement** | **Weighted %** |
| --- | --- |
| ≤6 months | 70 |
| >6 months but ≤1 year | 17 |
| >1 year but ≤2 years | 7 |
| >2 years but ≤5 years | 3 |
| >5 years | 3 |
| Never | <1 |
